# Analysis of HDL-microRNA panel in heterozygous familial hypercholesterolemia subjects with LDL receptor null or defective mutation

**DOI:** 10.1038/s41598-019-56857-2

**Published:** 2019-12-30

**Authors:** Roberto Scicali, Antonino Di Pino, Chiara Pavanello, Alice Ossoli, Arianna Strazzella, Antonia Alberti, Stefania Di Mauro, Alessandra Scamporrino, Francesca Urbano, Agnese Filippello, Salvatore Piro, Agata Maria Rabuazzo, Laura Calabresi, Francesco Purrello

**Affiliations:** 1 Department of Clinical and Experimental Medicine, University of Catania, Catania, Italy; 2 Centro E. Grossi Paoletti, Dipartimento di Scienze Farmacologiche e Biomolecolari, Università degli Studi di Milano, Milano, Italy; 3 Centro Dislipidemie, ASST Grande Ospedale Metropolitano Niguarda, Milano, Italy

**Keywords:** Predictive markers, Dyslipidaemias

## Abstract

In the last years increasing attention has been given to the connection between genotype/phenotype and cardiovascular events in subjects with familial hypercholesterolemia (FH). MicroRNAs (miRs) bound to high-density lipoprotein (HDL) may contribute to better discriminate the cardiovascular risk of FH subjects. Our aim was to evaluate the HDL-miR panel in heterozygous FH (HeFH) patients with an LDLR null or defective mutation and its association with pulse wave velocity (PWV). We evaluated lipid panel, HDL-miR panel and PWV in 32 LDLR null mutation (LDLR-null group) and 35 LDLR defective variant (LDLR-defective group) HeFH patients. HDL-miR-486 and HDL-miR-92a levels were more expressed in the LDLR-null group than the LDLR-defective group. When we further stratified the study population into three groups according to both the LDLR genotype and history of ASCVD (LDLR-null/not-ASCVD, LDLR-defective/not-ASCVD and LDLR/ASCVD groups), both the LDLR/ASCVD and the LDLR-null/not-ASCVD groups had a higher expression of HDL-miR-486 and HDL-miR-92a than the LDLR-defective/not-ASCVD group. Finally, HDL-miR-486 and HDL-miR-92a were independently associated with PWV. In conclusion, the LDLR-null group exhibited HDL-miR-486 and HDL-miR-92a levels more expressed than the LDLR-defective group. Further studies are needed to evaluate these HDL-miRs as predictive biomarkers of cardiovascular events in FH.

## Introduction

Atherosclerotic process is a progressive inflammatory disease caused by several external and hereditary factors^1^. Of these, the alterations of lipid and glucose metabolism play a key role in the pathogenesis and progression of atherosclerotic cardiovascular disease (ASCVD)^2,3^. In particular, an increase of the low-density-lipoprotein (LDL) cholesterol plasma level is causatively associated with ASCVD^4^. Despite changes in lifestyle and lipid lowering therapies, ASCVD is considered the principal condition of reduced quality-adjusted life years and death^5^. Thus, other mechanisms are involved in atherosclerosis in addition to LDL cholesterol.

Of note, in the last few years several studies have shown an important role of microRNAs (miRs) in the pathophysiology of atherosclerosis^6^. MiRs are a class of small noncoding RNAs that inhibit gene expression through the alteration of messenger RNA after transcriptional process into the cell^7^. Not all mature miRs are developed in the cell; thus, extracellular miRs such as plasmatic miRs may serve as intercellular messenger^8^. Many biological functions have been attributed to miRs; as concerns the metabolic pathways, they seemed to act as modulators of lipid metabolism, and transport and sustained inflammatory diseases^9,10^. In subjects characterized by high levels of LDL cholesterol such as familial hypercholesterolemia (FH), several studies demonstrated that a number of circulating miRs were upregulated from childhood, confirming their role in cholesterol homeostasis also in lipid genetic disorders^11,12^. To avoid plasmatic ribonucleases, miRs are transported by several carriers; in this context, the most crucial and stable transporters of miRs are plasmatic lipoproteins, especially the high-density lipoprotein (HDL) particles^13^.

Interestingly, Vickers *et al*. found that the HDL-miR panel was significantly different between homozygous familial hypercholesterolemia and normal subjects^14^.

In the last few years increasing attention has been given to the connection between genotype/phenotype and ASCVD in familial hypercholesterolemia^15–17^. In particular, several studies have shown that heterozygous FH (HeFH) subjects with an LDL receptor (LDLR) null mutation had an increased atherosclerotic burden with respect to HeFH subjects with an LDLR defective variant^18,19^. In this context, it may be helpful to evaluate the HDL-miR panel in HeFH subjects with a different LDLR genotype.

To better describe the link between HDL-miRs and HeFH genotype, in this study we aimed to investigate the plasma levels of several HDL-miRs correlated with lipid homeostasis and atherosclerotic pathway (miR-486, miR-92a, miR-24, miR-223, miR-625*, miR-122)^20,21^ in HeFH subjects with an LDLR null or defective mutation. Moreover, we evaluated the association of HDL-miRs with pulse wave velocity (PWV), an instrumental parameter of early atherosclerosis largely utilized in clinical practice for cardiovascular risk assessment^22^.

## Results

**Figure 1:**
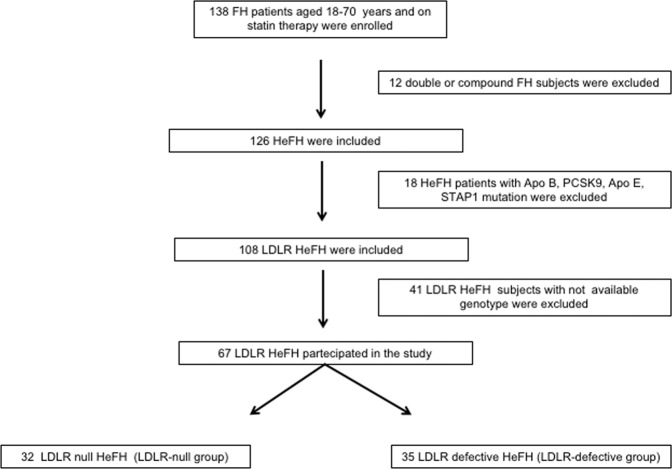
Enrollment of the Study Population. FH = familial hypercholesterolemia, HeFH = heterozygous familial hypercholesterolemia, LDLR = low-density lipoprotein receptor, ApoB = apolipoprotein B, PCSK9 = proprotein convertase subtilisin/kexin type 9, ApoE = apolipoprotein E, STAP1 = signal transducing adaptor family member 1.

In total, 138 FH patients were assessed and 67 LDLR HeFH patients satisfied the inclusion criteria and were involved in the study (Fig. 1). Partecipants were divided in two groups according to the LDLR genotype: 32 HeFH patients with an LDLR null mutation (LDLR-null group) and 35 HeFH patients with an LDLR defective mutation (LDLR-defective group).

The pretreatment lipid parameters of partecipants are displayed in Table 1. Of corse, the LDLR-null group had higher levels of total, LDL and non-HDL cholesterol than the LDLR-defective group.

**Table 1:** Pretreatment lipid values of the Study Population.

	LDLR-null group (n = 32)	LDLR-defective group (n = 35)	*p* Value between two groups
**Pretreated Lipid Values**			
Total cholesterol, mg/dL	387.62 ± 28.98	336.13 ± 29.44	<0.01
HDL cholesterol, mg/dL	51.58 ± 10.49	52.3 ± 11.23	0.85
Triglycerides, mg/dL	95.5 (71.5–150)	97.5 (71.75–150)	0.81
LDL cholesterol, mg/dL	282.32 ± 23.87	233.28 ± 22.43	<0.01
Non-HDL cholesterol, mg/dL	316.19 ± 37.92	265.83 ± 38.38	<0.01

Data are presented as mean ± standard deviation or median (interquartile range).

HDL = high-density lipoprotein, LDL = low-density lipoprotein, LDLR = low-density lipoprotein receptor.

The general features of partecipants are showed in Table 2. No discrepancy of metabolic parameters were showed in the two groups. Moreover, the values of systolic and diastolic BP and the percentage of smokers were the same in the LDLR-null and LDLR-defective groups. Furthermore, hs-CRP values were similar between the two groups. About medication, the LDLR-null group had a more protracted period of statin therapy than the LDLR-defective group (9.5 [2.5–17.5] vs 8 [1.5–12] years, *p* < 0.05). Furthermore, the majority of patients on ezetimibe were in the LDLR-null group than the LDLR-defective group (65.6% vs 40.0%, *p* < 0.05). Finally, similar proportions of antihypertensive medication were found between the two groups. In consideration of intensity of statin therapy, no subjects were on low-intensity statins. While a greater number of subjects on moderate-intensity statin were in the LDLR-defective group, the percentage of patients on high-intensity statin was more present in the LDLR-null group than the LDLR-defective group (75.0% vs 28.6%, *p* < 0.05). Moreover, the LDLR-null group had a greater PWV compared with the LDLR-defective group (9.58 ± 0.92 vs 7.41 ± 0.83 m/s, *p* < 0.05).

**Table 2:** Characteristics of the Study Population.

	LDLR-null group (n = 32)	LDLR-defective group (n = 35)	*p* Value between two groups
**Demographic Characteristics**			
N	32	35	
Age, years	48.69 ± 14.36	51.97 ± 15.47	0.37
Men, n (%)	18 (56.3)	19 (54.3)	0.61
Body mass index, kg/m^2^	25.21 ± 3.34	25.38 ± 3.79	0.84
ASCVD, n (%)	12 (37.5)	9 (25.7)	0.33
**Glucose Values**			
FPG, mg/dL	85.7 ± 6.84	88.11 ± 6.32	0.14
HbA1c, %	5.42 ± 0.29	5.51 ± 0.28	0.25
**Lipid Values**			
Total cholesterol, mg/dL	188.65 ± 25.26	186.5 ± 24.31	0.86
HDL cholesterol, mg/dL	49.16 ± 10.62	52.81 ± 8.84	0.07
Triglycerides, mg/dL	87 (80–117)	88.5 (81–119)	0.76
LDL cholesterol, mg/dL	121.54 ± 21.46	116.33 ± 20.48	0.31
Non-HDL cholesterol, mg/dL	140.48 ± 22.04	135.69 ± 22.07	0.16
ApoB, mg/dL	116.79 ± 25.02	110.53 ± 24.71	0.11
ApoAI, m g/dL	133.71 ± 23.34	137.62 ± 23.31	0.29
ApoB to ApoAI ratio	0.88 ± 0.34	0.81 ± 0.31	0.06
Lp(a), nmol/L	20.8 (10.05–62.95)	19.2 (9.69–41.05)	0.45
**Risk Factors**			
Systolic BP, mmHg	118.55 ± 9.33	119.17 ± 13.37	0.83
Diastolic BP, mmHg	70.48 ± 7.78	71.36 ± 9.17	0.28
Smoking, n (%)	10 (31.3)	13 (37.1)	0.61
hs-CRP, mg/dL	0.10 (0.05–0.19)	0.09 (0.04–0.15)	0.39
**Treatment**			
Duration of Statin therapy, years	9.5 (2.5–17.5)	8 (1.5–12)	<0.05
Ezetimibe, n (%)	21 (65.6)	14 (40.0)	<0.05
Antihypertensive therapy, n(%)	11 (34.4)	13 (37.1)	0.81
**Intensity of Statin Therapy**			
Low, n (%)	—	—	—
Moderate, n (%)	8 (25.0)	25 (71.4)	<0.05
High, n (%)	24 (75.0)	10 (28.6)	<0.05
**Early atherosclerotic biomarker**			
PWV, m/s	9.58 ± 0.92	7.41 ± 0.83	<0.05

Data are presented as mean ± standard deviation, percentages, or median (interquartile range).

ASCVD = atherosclerotic cardiovascular disease, FPG = fasting plasma glucose, HbA1c = glycated hemoglobin, HDL = high-density lipoprotein, LDL = low-density lipoprotein, LDLR = low-density lipoprotein receptor, ApoB = apolipoprotein B, ApoAI = apolipoprotein AI, Lp(a) = lipoprotein (a), BP = blood pressure, hs-CRP = high sensitivity C-reactive protein, PWV = pulse wave velocity.

**Figure 2:**
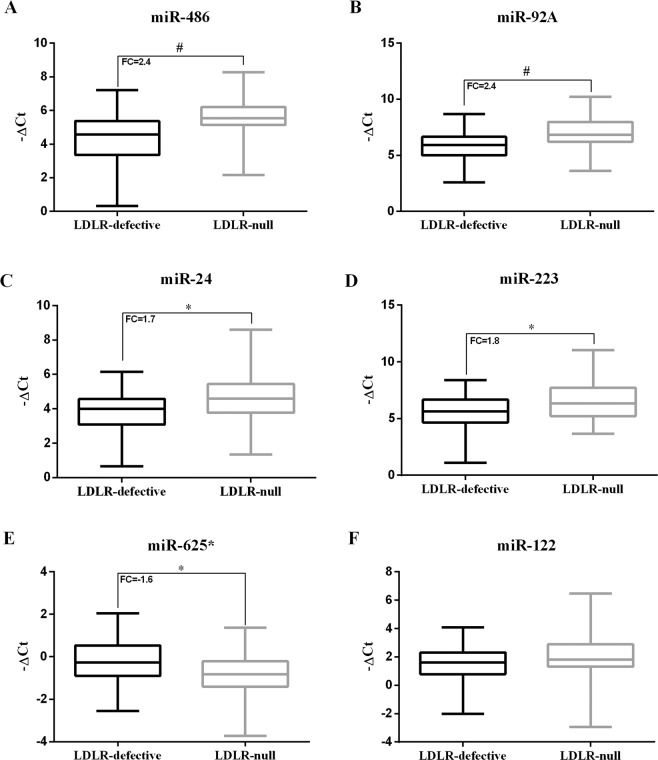
HDL-miR panel in the LDLR-null and the LDLR-defective groups. *p value < 0.05 versus LDLR-defective group, ^#^*p* value < 0.001 versus LDLR-defective group. To test differences of HDL-miR −ΔCt values in the two groups, Student’s t test was used.

**Figure 3:**
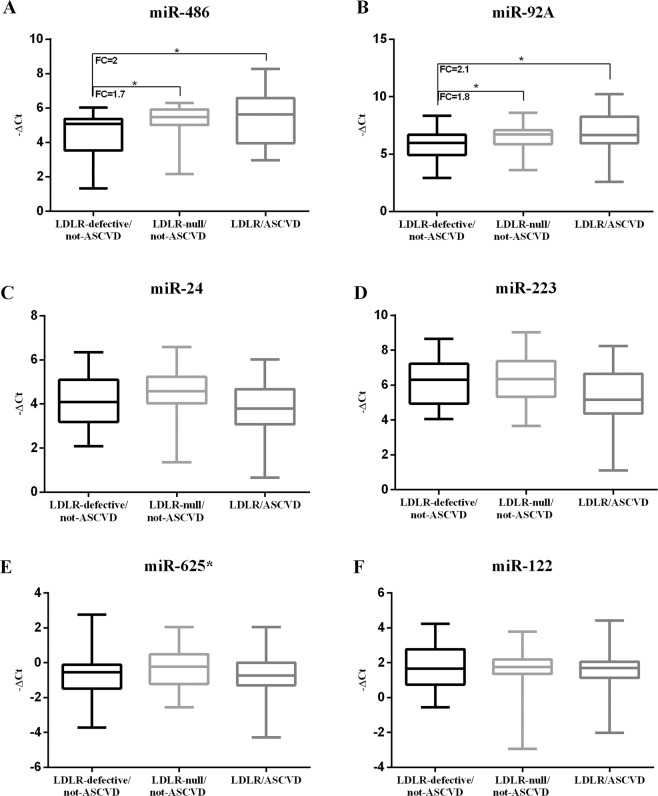
HDL-miR panel in the LDLR-null/not-ASCVD, LDLR-defective/not-ASCVD and the LDLR/ASCVD groups. *p value < 0.05 versus LDLR-defective/not-ASCVD group. To test differences of HDL-miR −ΔCt values in the two groups, Student’s t test was used.

Figure 2 reports the evaluation of HDL-miR panel in the two groups. While the level of HDL-miR-122 was similar between the two groups, a different expression of HDL-miR-486, HDL-miR-92a, HDL-miR-24, HDL-miR-223 and HDL-miR-625* was found between the two groups. In particular, HDL-miR-486 and HDL-miR-92a levels were largely represented in the LDLR-null group than the LDLR-defective group (HDL-miR-486 and HDL-miR-92a fold change +2.4 for both, *p* < 0.001 for both, Fig. 2A,B). Moreover, to compare the HDL-miR panel in FH patients with or without cardiovascular events, we performed another analysis and stratified the study population into three new groups according to both the LDLR genotype and history of ASCVD: LDLR-null/not-ASCVD group (20 FH patients), LDLR-defective/not-ASCVD group (26 FH patients) and LDLR/ASCVD group (21 FH patients) (Fig. 3).

Both the LDLR/ASCVD group and the LDLR-null/not-ASCVD group had a higher expression of HDL-miR-486 and HDL-miR-92a than the LDLR-defective/not-ASCVD group (for LDLR/ASCVD vs LDLR-defective/not-ASCVD group HDL-miR-486 and HDL-miR-92a fold change +2.0 and +2.1 respectively, *p* < 0.05 for both; for LDLR-null/not-ASCVD vs LDLR-defective/not-ASCVD group HDL-miR-486 and HDL-miR-92a fold change +1.7 and +1.8 respectively, *p* < 0.05 for both, Fig. 3A,B). Moreover, no difference of HDL-miR panel was found between LDLR/ASCVD and LDLR-null/not-ASCVD groups.

In the simple regression analysis, HDL-miR-92a and HDL-miR-486 were associated with PWV (r = 0.29, *p* < 0.01 for HDL-miR-92a and r = 0.27, *p* < 0.05 for HDL-miR-486). Subsequently, in the multiple regression analysis including these two HDL-miRs reaching significance and several cardiovascular risk factors as independent variables, both HDL-miR-92a and HDL-miR-486 remained significantly associated with PWV (*p* < 0.01 for both) (Table 3).

**Table 3:** Multiple regression analysis evaluating PWV as dependent variable.

Independent Variables	Coefficient β	*p* Value
Model^*^		
HDL-miR-92a, −ΔCt	0.343	<0.01
HDL-miR-486, −ΔCt	0.308	<0.01

^*^Model was adjusted for age, sex, smoking status, systolic blood pressure, LDL cholesterol and glycated hemoglobin.

## Discussion

The improved evaluation of genotype and phenotype of FH have recently focused on the impact of novel cardiovascular risk biomarkers in FH subjects^23,24^. In this study, we examined the role of HDL-miRs in HeFH subjects with an LDLR null or defective mutation; to our learning, no other studies explored HDL-miR panel in these FH subgroups. We showed that HDL-miR-486 and HDL-miR-92a were more expressed in the LDLR-null group than the LDLR-defective group; moreover, we found that both the LDLR-ASCVD group and the LDLR-null group had significant HDL-miR-486 and HDL-miR-92a levels compared with the LDLR-defective group. Finally, we showed a significant association between HDL-miR-486, HDL-miR-92a and PWV. Our findings are in line with previous studies showing a possible role of these two miRs in lipid homeostasis and the atherosclerotic process; in fact, Liu D. *et al*. found that miR-486 promoted cholesterol concentration in macrophage-derived foam cells^25^. Moreover, Zhang *et al*. showed that miR-486 was higher in patients with acute myocardial infarction than controls^26^. Liu F. *et al*. found that also miR-92a was higher in patients with coronary artery disease than controls^27^; furthermore, several studies showed the possible role of miR-92a in the atherosclerotic process by the promoting endothelial dysfunction and apoptosis in cardiomyocytes^28–30^. In line with these findings, Niculescu *et al*. found that miR-486 and miR-92a levels in HDL subfractions may identify subjects at increased risk of recurrent cardiovascular events^31^; moreover, they showed that the inhibition of miR-486 and miR-92a decreased liver and plasma cholesterol levels by restoring the lipid metabolism liver genes such as ATP binding cassette subfamily G member 4 (ABCG4) and the sterol regulatory element binding transcription factor-1 (SREBF1)^32^. In this context, miR-486 and miR-92a linked to HDL may be important modulators of HDL functions such as cholesterol efflux, endothelial nitric oxide synthase (eNOS) and nitric oxide (NO) production^33^.

FH patients are characterized by an increased risk of cardiovascular events^34^; however, ASCVD risk is not the same in FH population. In particular, several studies demonstrated that HeFH patients with an LDLR null mutation had a greater atherosclerotic burden than the HeFH patients with an LDLR defective variant^18,19,35^; this is probably due to the higher cholesterol burden of LDLR null HeFH compared with LDLR defective HeFH. In our study, all FH patients were on statin therapy and lipid values were similar between the two groups. It is well known that statin treatment has significantly reduced the risk of ASCVD in FH subjects^36,37^. However, despite statin therapy, the LDLR null HeFH patients have a higher ASCVD risk compared with the LDLR defective HeFH patients on statin therapy^38^. In line with this consideration, in our study we found that the LDLR null HeFH patients had a higher PWV than the LDLR defective HeFH patients. Moreover, in our population we showed a direct association of HDL-miR-486 and HDL-miR-92a with PWV; thus, these miRs may support the increase of cardiovascular risk and the advance of atherosclerosis in addition to LDL cholesterol burden in FH patients.

Recent clinical trials where increased levels of HDL cholesterol have been obtained, failed to demonstrated a significant reduction of ASCVD in a large population at high cardiovascular risk^39,40^. These findings strongly support the hypothesis of “HDL quality” and not HDL quantity. Several studies have focused on the alterations of HDL quality in FH subjects, and, in particular, on their decreased ability to support cholesterol efflux from macrophages, and on their diminished anti-inflammatory and anti-oxidant properties^41–43^. In this context, miRs bound to HDL particles may effectively modulate their function on lipid metabolism and the atherosclerotic process. Recently, several studies have focused attention on miRs as possible targets in FH patients^33,44^. However, no prospective studies have shown if HDL-miR-486 and HDL-miR-92a could be good predictors for subsequent cardiovascular events in FH subjects; thus, additional studies are required to investigate their role in the progression of the atherosclerotic burden and their possible relationship with ASCVD in a large FH population.

There are several limitations to our study. First, we are unable to establish a causal relationship and temporality between HDL-miR-486 and HDL-miR-92a and possible changes in PWV by the cross-sectional design of this study. The number of studied patients was relatively small; however, we showed a significant difference of HDL-miR panel in the groups and an independent association of HDL-miR-486, HDL-miR-92a and PWV was found. Finally, other cardiovascular parameters such as cholesterol burden were not available and, thus, were not taken into consideration.

In conclusion, HDL-miR-486 and HDL-miR-92a levels were more expressed in the LDLR-null group than the LDLR-defective group; moreover, HDL-miR-486 and HDL-miR-92a were significantly associated with PWV. Our study suggests that these miRs may be helpful to improve cardiovascular risk stratification; additional studies are required to investigate HDL-miRs as predictive biomarkers of cardiovascular events and possible treatment targets in FH patients.

## Methods

### Study design and population

It was an observational study in subjects with a diagnosis of FH already confirmed by genetic evaluation^45,46^. The patients were evaluated from the University Hospital of Catania and the Dyslipidemia Center of the Niguarda Hospital in Milan, Italy, two tertiary lipid centers, from April 2017 to December 2018^16^. The age of partecipants were over 18 and under 70 years and assumed statin therapy at the moment of the study. In particular, we only included FH patients with a null or defective LDLR genetic variant; the reason of this restricted criteria was that LDLR mutations were associated with several genotype/phenotype patterns that could contribute to the heterogeneity of FH population. In this context, we aimed to evaluate possible novel cardiovascular biomarkers such as HDL-miRs in these subjects.

The study was accepted by the local ethics committees Catania 2 and Milano Area 3 in accordance with the ethical standards of the institutional and national research committees and with the 1964 Declaration of Helsinki and its later amendments or comparable ethical standards. Informed consent was attained from all partecipants the study.

All subjects obtained a physical examination and clinical biochemistry parameters measured as previously described^2^. Anthropometric parameters, glycemic status, arterial pressure, medications, ASCVD, advanced renal disease and smoking abits were defined as previously described^22,47,48^. The severity of liver disease was defined as previously described^49^. The exclusion criteria were defined as previously described^22^.

### Biochemical analysis

Fasting plasma glucose (FPG), serum total cholesterol, TG, high-density lipoprotein (HDL) cholesterol, hs-CRP, Apolipoprotein B (ApoB), Apolipoprotein A1 (ApoA1) were assessed as previously described^50^. Levels of lipoprotein (a) [Lp(a)] were measured as previously described^22^. LDL cholesterol was obtained by the Friedewald formula. Glycated hemoglobin (HbA1c) was measured as previously described^51^.

### HDL purification

HDL particles (d = 1.063–1.21 g/mL) were obtained by sequential ultracentrifugation from plasma collected from all subjects. After separation, HDL particles were dialyzed against sterilized PBS to remove the high-salt KBr solutions and immediately frozen at −80 °C until use^52^. By using *ExoTEST Ready to Use Kit for ELISA Exosome quantification* (*Hansa BioMed*), we assessed that the purified HDL particles were negative for the classic exosomal protein marker CD9.

### RNA extraction

HDL-carried miRs were extracted from 400 μl of purified HDL particles as previously described^53^.

### Single taqMan assays

MiR expression levels were evaluated by *Single TaqMan MicroRNA Assays* as previously described^53^.

### Pulse wave velocity evaluation

PWV was performed as previously described^54^.

### Statistical analysis

The Kolmogorov-Smirnov test was used to assess normality distribution of all variables. Continuous parametric and non parametric data are described as mean ± standard deviation (SD) or median (interquartile range-IQR), respectively; moreover, categorical variables are reported as frequency (percentage) and evaluated by χ^2^ test. Continuous non-parametric variables (TG, Lpa, duration of statin therapy and hs-CRP) were logarithmically transformed for statistical analysis to diminish dissymetry. MiR expression data are presented as minus Delta Ct values (−ΔCt), calculated according to the following formula: −1*(Threshold Cycle of analysed miRNA − Threshold Cycle of U6 in each sample). MiR expression Fold Changes (FC) were calculated by applying the 2^−ΔΔCT^ method by using small nuclear RNA U6 as reference gene. Student’s t test was performed for clinical and biochemical characteristics. Simple regression analysis was used to evaluate the relation of HDL-miRs with PWV. Subsequently, to analyze a possible independent association with changes of PWV, HDL-miRs reaching significance were included in a multivariate analysis with principal cardiovascular risk factors (age, sex, smoking, systolic BP, HbA1c, and LDL cholesterol). The variance inflation factor (VIF) was performed to assess the problem of multicollinearity in multivariate analysis. All statistical analyses were obtained by IBM SPSS Statistics for Windows version 23. For all tests, *p* < 0.05 was considered significant.

### Ethical approval

This study was approved by the local ethics committee in accordance with the ethical standards of the institutional and national research committees and with the 1964 Declaration of Helsinki and its later amendments or comparable ethical standards. This article does not contain any studies with animals performed by any of the authors.

### Informed consent

Informed consent was obtained from each participant enrolled in the study.
